# The LUCID study: living with ulcerative colitis; identifying the socioeconomic burden in Europe

**DOI:** 10.1186/s12876-021-02028-5

**Published:** 2021-12-04

**Authors:** Leonardo Ruiz-Casas, Jonathan Evans, Alison Rose, Gabriel Ghizzi Pedra, Alan Lobo, Alan Finnegan, Bu Hayee, Laurent Peyrin-Biroulet, Andreas Sturm, Johan Burisch, Helen Terry, Luisa Avendano, Seb Tucknott, Gionata Fiorino, Jimmy Limdi

**Affiliations:** 1 HCD Economics, The Innovation Centre, Keckwick Lane, Daresbury, Warrington, England, UK; 2 Sheffield Teaching Hospitals NHS Foundation Trust, Sheffield, UK; 3 The University of Sheffield, Sheffield, UK; 4 University of Chester, Chester, UK; 5 King’s College Hospital NHS Foundation Trust London, London, UK; 6 University Hospital Centre Nancy, Nancy, France; 7 DRK Kliniken Berlin, Berlin, Germany; 8 Hvidovre Hospital, Gastrounit, Hvidovre, Denmark; 9 Crohn’s and Colitis UK, Hatfield, Hertfordshire, UK; 10 European Federation of Crohn’s and Ulcerative Colitis Associations, Brussels, Belgium; 11 IBDrelief, Brighton, UK; 12 Istituto Clinico Humanitas, Milan, Italy; 13 Pennine Acute Hospitals NHS Trust, Manchester, UK

**Keywords:** Ulcerative colitis, Health-related quality of life, Socioeconomic burden

## Abstract

**Background:**

Ulcerative colitis (UC) is an inflammatory bowel disease with increasing prevalence worldwide. Current treatment strategies place considerable economic and humanistic burdens on patients. The aim of this study was to determine the socioeconomic burden of UC in adult patients in European countries in a real-world setting.

**Methods:**

In this retrospective, cross-sectional and observational pan-European study, patients with moderate or severe UC were assigned to ARM 1 and patients who had moderate or severe UC but achieved mild or remission status 12 months before index date (or clinical consultation date), were assigned to ARM 2. Clinical and medical resource use data were collected via electronic case report forms, and data on non-medical and indirect costs, and health-related quality of life (HRQoL) were collected via patient and public involvement and engagement (PPIE) questionnaires. Per-patient annual total costs per ARM and per country were calculated using the collated resource use in the last 12 months (between the start of the documentation period and patient consultation or index date) and country specific unit costs. Quality of life was described by arm and by country.

**Results:**

In the physician-reported eCRF population (n = 2966), the mean annual direct medical cost was €4065 in ARM 1 (n = 1835) and €2935 in ARM 2 (n = 1131). In the PPIE population (ARM 1, n = 1001; ARM 2, n = 647), mean annual direct cost was €4526 in ARM 1 and €3057 in ARM 2, mean annual direct non-medical cost was €1162 in ARM 1 and €1002 in ARM 2, mean annual indirect cost was €3098 in ARM 1 and €2309 ARM 2, and mean annual total cost was in €8787 in ARM 1 and €6368 in ARM 2. HRQoL scores showed moderate to high burden of UC in both groups.

**Conclusions:**

The cost and HRQoL burden were high in patients in both ARM 1 and ARM 2 indicating unmet needs in the UC active population.

**Supplementary Information:**

The online version contains supplementary material available at 10.1186/s12876-021-02028-5.

## Background

Ulcerative colitis (UC) is a chronic, relapsing and remitting and potentially progressive form of inflammatory bowel disease (IBD) of uncertain aetiology, characterised by inflammation localised in the mucosa of the rectum and colon [1–3]. UC can lead to disease complications (strictures, bowel perforations and toxic megacolon, among others) and extraintestinal manifestations (EIM) in other tissues and organs, including the skin, joints, eyes, mouth, liver and lungs [4, 5]. It has an estimated incidence of 1.2–20.3 per 100,000 and a prevalence of 7.6–245 per 100,000 [6–10].

Current UC treatment strategies include medications that aim to induce and maintain clinical remission, prevent complications (such as hospitalisation, surgery, colorectal cancer and EIM) and improve health-related quality of life (HRQoL) [4, 11]. Treatment options are usually dictated by severity of disease and patient preference. In most cases, a step-up medication strategy is followed; patients with mild to moderate UC symptoms are usually treated with 5-aminosalicylates and corticosteroids, whereas those with moderate to severe symptoms are treated with corticosteroids, immunosuppressants (such as thiopurines) and more advanced targeted therapies such as monoclonal antibodies and more recently an orally administered JAK inhibitor [2–4]. Despite an expanding armamentarium of therapeutic options, the fluctuating course of UC and often unpredictable response to treatment implies that patients often experience disease “flares” requiring urgent out-patient consultations, hospitalisation and surgery for symptoms driven by refractory inflammation, complications of disease and hitherto under-recognised morbidity from chronic pain and psychosomatic issues [12–18].

Exacerbation of UC symptoms has a significant impact on health-care utilisation with annual costs of UC care reported to be as high as USD 8.1–14.9 billion in the USA and Euros 12.5–29.1 billion in Europe. In 2006, the mean annual expenditure on healthcare for UC across several European countries was estimated to be €1524 per patient, and the most expensive costs were medical and surgical hospitalisations accounting for 45% of the total expenditure [19]. Frequent health care utilisation and active UC (periods of high intensity of symptoms) contributes to work absenteeism and disability, with a significant negative impact on HRQoL [19, 20]. Furthermore, fatigue, feeling of isolation and loss of control may also negatively impact of quality of life and psychosocial well-being of patients and indeed health-care utilisation through direct and indirect costs of care [12–15, 17, 18, 21, 22].

Despite increasing awareness of economic and humanistic burdens of UC, there is a lack of extensive, up-to-date and real-world information on the socioeconomic burden of active UC.

The overall aim of this study was to provide robust evidence for the identification of the overall socioeconomic burden of UC in the EU5 (France, Germany, Italy, Spain, United Kingdom [UK]), Denmark, Norway, Poland, Romania and Turkey in a real-world setting.

The primary objective of this study was to explore and quantify the annual costs of living with active UC from a societal perspective (including direct medical, non-medical and indirect costs), and the secondary objective was to explore the effect of UC on the HRQoL and productivity using patient-reported outcomes measurements (PROMs) and work-related activity. Patient stratification by country and by disease severity added granularity to the analyses.

## Methods

### Study design

This was a non-interventional, descriptive, retrospective, cross-sectional, pan-European (EU5, Denmark, Norway, Poland, Romania, and Turkey) multi-site study. Gastroenterologists were recruited over the study period between August 2018–February 2019. Each gastroenterologist provided data from the medical records of their patients using standardised questionnaires called electronic Case Report Forms (eCRFs) for up to 10 eligible patients with UC per ARM (20 in total).

### Participants

**Fig. 1:**
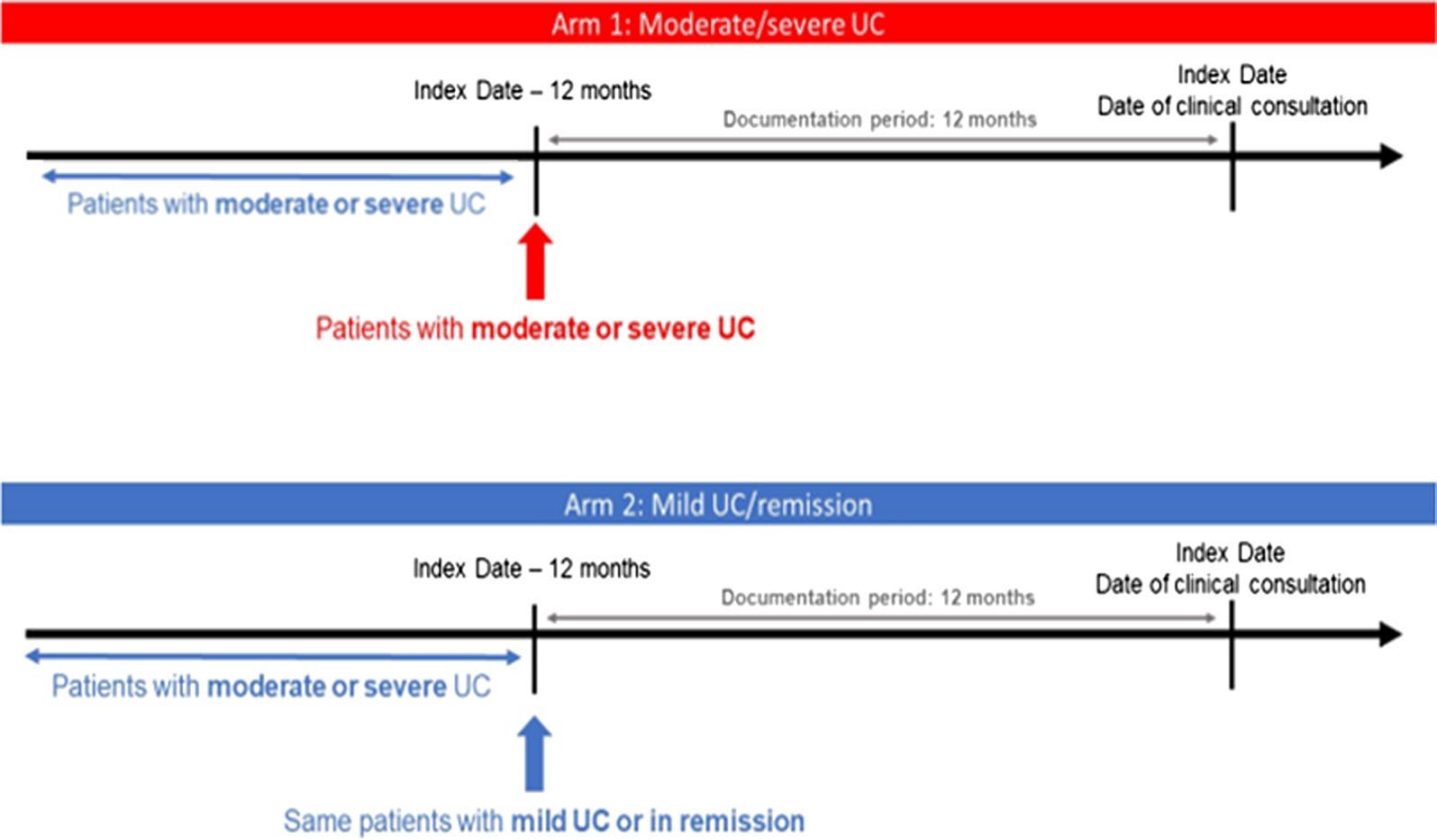
A representation of the study design, indicating the index date, documentation period and ARMs of study. *UC* Ulcerative colitis

The primary and secondary objectives were analysed by disease severity (two ARMs of the study) and country. The index date was defined as the date of clinical consultation between the patient and the participating physician. ARM 1 included patients with moderate or severe UC at initiation of the documentation period (12 months prior to the index date) as indicated by the Mayo score, simple clinical colitis activity index (SCCAI) scores, or physician global assessment for UC. ARM 2 included patients with moderate or severe UC 24 months prior to the index date that achieved mild UC or remission at initiation of documentation period (12 months prior to the index date) as indicated by Mayo score or SCCAI scores for UC, or physician global assessment (Fig. 1). Clinical remission was defined as a Mayo score of 0–2, PGA of 0 or SCCAI < 2. Mild UC was defined as a Mayo score of 3–5, PGA of 1 or SCCAI 2–4. Moderate-severe disease was defined by as a Mayo score of 6–10 (moderate) and 11–12–9 (severe), PGA of 2(moderate), PGA 3(severe) or SCCAI > 5. ARM 1 and ARM2 definitions were independent of the fact that patient’s severity status could fluctuate within the documentation period (as this was the case in both arms, due to the fluctuant nature of the disease).

### Patient inclusion criteria

Patients were included in the study if they were 18 years or older at the index date, with UC diagnosis confirmed by endoscopy and histology at least 24 months before the index date. Patients in ARM 1 had moderate or severe UC at initiation of documentation period (12 months prior the index date) as indicated by Mayo or Simple Clinical Colitis Activity Index (SCCAI) scores (between 6 and 10 for moderate patients, and score between 11 and 12 for severe patients), or by physician global assessment. Patients in ARM 2 had moderate or severe UC that achieved mild UC or remission at initiation of documentation period (12 months prior the index date), where mild UC was indicated by Mayo or simple clinical colitis activity index (SCCAI) scores (3–5), or by physician global assessment and clinical or endoscopic remission was indicated by Mayo or SCCAI scores (less than 2) or by physician global assessment. Furthermore, patients were required to be able to read, understand and sign the informed consent form in their local language.

### Patient exclusion criteria

Patients were excluded from the study if they were diagnosed with Crohn’s Disease and/or IBD unclassified, initiated clinical trials/non-interventional study for IBD and/or UC treatment 12 months prior the index date, could not understand the PPIE questionnaire for issues such as language barriers or suffered from a physical or mental condition that prevented them from providing informed consent.

### Variables

Gastroenterologists provided data on each patient, that included demographic, clinical (disease history, diagnosis, symptoms, complications, etc.) and economic information (direct medical resource utilisation: medications, consultations, hospitalisations and surgical interventions, tests and procedures used for diagnosis and follow-up of disease), via the electronic case report form.

To capture data on HRQoL and other UC-related costs, gastroenterologists invited patients to provide information via patient and public involvement and engagement (PPIE) questionnaires. Data on HRQoL were captured via validated tools such as EuroQol-5D-5L (EQ-5D) [23, 24], the inflammatory bowel disease disability index (IBD-DI) [25] and the IBD control questionnaire (IBD Control Q; UK only) [26]. The work productivity and activity impairment (WPAI) [27] questionnaire were used to capture productivity losses and impairment in daily life activities due to their UC condition.

Data on UC-related costs included direct non-medical costs (professional caregiving, alternative therapies, aids and home equipment/adaptations, transportation and transfer payments—including state benefits or disability allowances) and indirect costs (loss of wages and productivity for patients and their carers due to absenteeism or impairment while at work, including early retirement or long-term sick leaves due to UC).

### Data sources

Clinical health resource use data for each patient was captured in the eCRF while direct non-medical and indirect resource use as well HRQoL was captured in the PPIE. National unit costs for each resource item were sourced from country-specific sources, governmental documents and national drug pricing databases. A list of used sources for each country is available in Additional file 1: Table 1.

### Statistical analysis

To calculate the cost per ARM, the resource use (RU) and unit cost data were collated using homogeneous collection methods (both CRF and PPIE questionnaires were equal across countries, with some country specific differences to account for differences in health care systems) to ensure international comparability, followed by applying unit costs to RU data for each country.

Per-patient costs for the 12 months between the start of the documentation period and patient consultation or index date were calculated by multiplying the quantities of the resource used with the national unit price of each resource (updated to 2019 prices using inflation indicators via http://ec.europa.eu/eurostat/web/hicp/data/database). Applying the unit costs was performed via a transparent model design using simple formulae: Price_i_ × Quantity_i_ = Cost_i_; i = 1–n (n = number of cost items). Country-specific unit costs were multiplied with the resource use quantity to calculate the mean per-patient UC-related cost for the 12 months prior to index date. All local currency total costs were converted to Euros using the official conversion rates as of 30^th^ of august 2019 (https://www.oanda.com/currency/converter/).

Annual Indirect costs were computed by assigning an opportunity cost (average salaries of the different countries were considered) to the disease-related productivity losses of patients and informal carers. All descriptive analyses were performed using Stata 16.

### Ethics

The study was approved by the Research Ethics Sub Committee of the Faculty of Health and Social care within the University of Chester (UoC). This study complies with UK national requirements and followed the Guidelines for Good Pharmacoepidemiology Practices (GPP) [28]. An Expert Review Group (ERG) was established to ensure the maintenance of quality standards and provide overall study guidance on behalf of HCD Economics, UoC, charity partner Crohn’s and Colitis UK (CCUK) and funding sponsors.

## Results

### Study population

The 379 recruited gastroenterologists, primary care and internal medicine practitioners completed electronic CRFs for 2979 patients. Amongst these, 1657 (55%) patients completed the PPIE which was matched to the corresponding CRF. Patient response rates for the completion of PPIEs questionnaires ranged from 16% in the United Kingdom to 95% in Romania (see Additional file 1: Table 2 for CRF and PPIE response by country), which was a reflection of the voluntary nature of the PPIE completion and patient willingness to contribute (no incentives were given to patients for the completion).

Out of the 2979 CRFs, 1835 were assigned to ARM 1, 1131 were assigned to ARM 2, and 13 could not be placed into an ARM due to lack of disease severity data. Disease severity data reflected the fluctuant nature of UC activity, with 39% of patients in ARM 1 improving to remission or mild status, and 23% of patients in ARM 2 worsening to moderate or severe status. Gender, age and body mass index (BMI) were largely similar across the two arms (Table 1).

**Table 1:** Study population demographics

	ARM 1	ARM 2	Overall
**Number of patients (%)**	1835 (62)	1131 (38)	2966 (100)
Male (%)	1023 (56)	615 (53)	1638 (55)
Female (%)	812 (44)	516 (47)	1328 (45)
**Number of severities (12 months prior index date)**	1835	1131	2966
Remission (%)	–	520 (46)	520 (17)
Mild (%)	–	611 (54)	611 (21)
Moderate (%)	1457 (79)	–	1457 (49)
Severe (%)	378 (21)	–	378 (13)
**Number of severities (last recorded)**	1758	1104	2862
Remission (%)	134 (8)	300 (27)	434 (15)
Mild (%)	545 (31)	547 (50)	1092 (38)
Moderate (%)	885 (50)	209 (19)	1094 (38)
Severe (%)	194 (11)	48 (4)	242 (8)
**Age (n)**	1835	1131	2966
Mean (SD)	46 (15)	48 (15)	47 (15)
**BMI (n)**	1835	1,131	2966
Mean (SD)	24.06 (4.2)	24.38 (3.8)	24.18 (4.1)
**Country (n)**	1835	1131	2966
Denmark (%)	9 (36)	16 (64)	25 (100)
France (%)	336 (67)	163 (33)	499 (100)
Germany (%)	256 (76)	79 (24)	335 (100)
Italy (%)	348 (62)	217 (38)	565 (100)
Norway (%)	12 (57)	9 (43)	21 (100)
Poland (%)	132 (62)	82 (38)	214 (100)
Spain (%)	344 (58)	250 (42)	594 (100)
Turkey (%)	42 (35)	78 (65)	120 (100)
UK (%)	256 (54)	219 (46)	475 (100)
Romania (%)	100 (85)	18 (15)	118 (100)

*BMI* body mass index, *n* number, *SD* standard deviation, *UK* United Kingdom

Of the 1657 (55%) patients who completed the PPIE questionnaire, 1001 were assigned to ARM 1, 647 were assigned to ARM 2 and 9 could not be placed into an ARM due to lack of severity data.

### Diagnosis and monitoring

Most patients in ARM 1 and ARM 2 of the eCRF population had left-sided colitis (40% and 43%, respectively), followed by extensive colitis (26% and 21%, respectively). The mean age of diagnosis was 40.5 years across both ARMs. Across countries, the mean age at diagnosis varied from 35.8 years in Poland to 45.3 years in Italy.

### Comorbidities

In both arms of the eCRF population, 71% of patients experienced at least one comorbidity; anaemia was the most commonly reported in 34% of patients (with 38% and 27% in ARMS 1 and 2 respectively), followed by anxiety and depression in 31% and 16% of patients.

### Disease activity

Patients experienced various symptoms at index date and 12 months prior, where the main five symptoms were diarrhoea (62% and 55% at 12 months prior index date, and 43% and 30% at index date in ARM 1 and ARM 2 respectively), rectal bleeding (55% and 44% at 12 months prior index date, and 28% and 14% at index date in ARM 1 and ARM 2 respectively), anaemia (54% and 41% at 12 months prior index date, and 31% and 19% at index date in ARM 1 and ARM 2 respectively), cramping pain (47% and 37% at 12 months prior index date, and 32% and 20% at index date in ARM 1 and ARM 2 respectively) and tiredness/fatigue (38% and 42% at 12 months prior index date, and 34% and 36% at index date in ARM 1 and ARM 2 respectively).

UC-related complications in patients from ARM 1 and ARM 2 were reported throughout their life, with strictures being the most frequently reported complications (12% and 9% respectively), followed by fistulas (10% and 7% respectively) and perforations (4% and 3% respectively).

Despite patients in ARM 2 achieving mild or remission status at the initiation of the documentation period, the severity of symptoms and complications remained relatively high both at that timepoint but also at index date.

Patients in ARM 1 and ARM 2 experienced anaemia (69% and 63%, respectively) and EIMs such as joint inflammation (20% in both ARMs) and mouth ulcers (13% and 19%, respectively).

### Treatment history

To relieve the symptoms of UC, conventional non-biologic and biologic therapies were typically prescribed. Non-biologic therapies included mesalazine (62% in ARM 1 and 68% in ARM 2), azathioprine (18% in ARM 1 and 12% in ARM 2) and prednisone (15% in ARM 1 and 8% in ARM 2), but also less commonly prescribed therapies such as sulfasalazine (9% of all patients), budesonide (8%), methotrexate (4%) or tacrolimous (1%). Biologic treatments were prescribed to 22% (n = 652) of the study population, with infliximab (49% in ARM 1 and 54% in ARM 2 respectively) and adalimumab (38% and 35%) being the most common, and vedolizumab (10% and 8%), golimumab (9% and 6%) and others (not specified, 2% and 1%) less commonly prescribed.

Five percent (n = 153) of all the patients had undergone at least one surgery for their UC (with 5% and 6% of patients of ARM1 and 2 respectively) being colectomy with ileostomy the most frequent procedure (29% of these patients) followed by colectomy with ileorectal anastomosis (21%), ileal pouch-anal anastomosis (15%), proctocolectomy with permanent ileostomy (12%), with an additional 25% of patients that received “other” procedures. An additional 6% (n = 168) of the patients in the LUCID study sample were considered “candidates for surgery”, with 6% and 4% of patients in ARM 1 and 2 respectively.

## Costs

Table 2 summarises total direct, direct non-medical and medical costs in ARMs 1 and 2.

**Table 2:** Total direct medical, direct non-medical and indirect costs by ARM

	ARM 1	ARM 2	Overall
**CRF population, direct medical cost (n)**	1835	1131	2966
**Mean (SD)**	€4065 (10,182)	€2935 (7327)	€3634 (9214)
Biologic Tx	€2646 (9285)	€1829 (6979)	€2334 (8488)
Procedure/Test	€174 (215)	€174 (204)	€174 (211)
Non-Biologic Tx	€376 (1692)	€358 (1437)	€369 (1599)
Surgery	€87 (638)	€96 (615)	€90 (629)
Consultation	€492 (859)	€402 (535)	€458 (753)
Hospitalisation	€289 (1885)	€76 (571)	€208 (1527)
**PPIE population, (n)**	1001	647	1648
**Direct medical cost, mean (SD**)	€426 (10,849)	€3057 (7790)	€3949 (9787)
Biologic Tx	€3007 (9718)	€1964 (7468)	€2,597 (8915)
Procedure/Test	€171 (193)	€181 (177)	€175 (187)
Non-Biologic Tx	€396 (1780)	€394 (1426)	€395 (1650)
Surgery	€74 (471)	€63 (403)	€70 (445)
Consultation	€527 (1064)	€387 (535)	€472 (897)
Hospitalisation	€352 (2238)	€67 (649)	€240 (1796)
**Direct non-medical cost, mean (SD)**	€1162 (3761)	€1002 (3276)	€1099 (3578)
Professional caregiver	€493 (3326)	€437 (2581)	€471 (3054)
Home alteration	€48 (187)	€41 (194)	€45 (190)
OTC medication	€80 (153)	€64 (124)	€74 (142)
Transport	€96 (196)	€116 (584)	€104 (397)
Transfer payments	€218 (1134)	€129 (890)	€183 (1045)
Alternative therapy	€227 (737)	€216 (866)	€222 (790)
**Indirect cost, mean (SD)**	€3098 (9091)	€2309 (7379)	€2789 (8467)
Non-professional caregiver	€767 (3689)	€642 (2791)	€718 (3365)
Retire/stop working	€1456 (6724)	€1419 (6330)	€1441 (6570)
Time off work in the last 12 months	€875 (3311)	€249 (1112)	€630 (2689)
**Total cost PPIE Pop only, mean (SD)**	€8787 (15,793)	€6368 (12,149)	€7837 (14,517)

*CRFs* case record forms, *n* number *PPIEs* patient public involvement engagement, *SD* standard deviation, *Pop* population

### Direct medical costs (via eCRF)

Overall mean direct medical costs were €3949 in the PPIE population (n = 1648) and €3634 in the CRF population (n = 2966) (Table 2).

In the eCRF population, the mean direct medical cost was €4065 in ARM 1 (n = 1835) and €2935 in ARM 2 (n = 1132). The mean cost was highest in Norway (€5373), Germany (€5320), Denmark (€4851) and Italy (€4307), and lowest in Turkey (€1299) (Table 3). Biologic treatments incurred the highest mean costs across ARMS 1 and 2 and in every country, except for Turkey, where costs for consultations were higher than biologic treatments.

**Table 3:** Total direct medical, direct non-medical and indirect costs by country

	Denmark (n = 31)	France (n = 500)	Germany (n = 335)	Italy (n = 565)	Norway (n = 22)	Poland (n = 214)	Spain (n = 595)	Turkey (n = 120)	United Kingdom (n = 479)	Romania (n = 118)	Overall (n = 2979)
**CRF population, direct medical cost (n)**	31	500	335	565	22	214	595	120	479	118	2979
Mean (SD)	€4851 (12,911)	€3357 (5841)	€5320 (13,311)	€4307 (11,414)	€5373 (13,269)	€3762 (8740)	€4084 (9418)	€1299 (7700)	€2024 (4505)	€2792 (6797)	€3643 (9217)
**PPIE population, (n)**	23	334	167	318	6	128	431	57	81	112	1657
Direct medical cost, mean (SD)	€5362 (14,754)	€3303 (5777)	€4802 (13,142)	€4500 (12,100)	€2404 (4822)	€2787 (6380)	€4551 (10,189)	€2478 (11,057)	€3444 (7077)	€2903 (6960)	€3945 (9773)
Direct non-medical cost, mean (SD)	€220 (486)	€796 (2102)	€728 (4556)	€1787 (3965)	€560 (591)	€1318 (4518)	€1321 (4115)	€63 (164)	€775 (2841)	€429 (1668)	€1096 (3570)
Indirect cost, mean (SD)	€638 (2360)	€4334 (13,336)	€2210 (7252)	€2876 (6878)	€0 (0)	€1964 (5055)	€3061 (7726)	€80 (414)	€3045 (8001)	€820 (2202)	€2812 (8500)
Total cost, mean (SD)	€6219 (15,925)	€8434 (15,178)	€7741 (16,357)	€9162 (16,921)	€2964 (4782)	€6069 (9769)	€8934 (14,355)	€2620 (11,041)	€7264 (12,783)	€4152 (7214)	€7854 (14515)

*PPIE* patient and public involvement and engagement, *SD* standard deviation

### Direct non-medical (via PPIE)

Mean annual direct non-medical cost was €1162 in ARM 1 and €1002 in ARM 2 (Table 2). The highest overall direct non-medical costs across all countries were observed in the EU5 countries and Poland; the largest was reported in patients from Italy (€1787) (Table 3). Professional caregiver costs were the most expensive items across both ARMS and most countries, followed by support services (e.g., nutritionists and physiotherapists) and transfer payments from the government.

### Indirect costs (via PPIE)

Mean annual indirect cost was €3098 in ARM 1 and €2309 ARM 2 (Table 2). Most indirect costs were attributed to long term sick leaves and early retirements from patients, with close to 50% of total indirect costs, whereas time from informal caregivers and time off work in the last 12 months had similar proportions. At the country level, France had the highest recorded mean indirect cost (€4,334) followed by Spain (€3061), the UK (€3045) and Italy (€2876) (Table 3).

### Calculating HRQoL via validated PROMs

Tables 4, 5 and 6 summarise PPIE population patient responses to the EQ-5D-5L, IBD-DI and WPAI questionnaires.

**Table 4:** Patient-reported IBD-DI, EQ-5D and WPAI scores in the PPIE population by ARM

	ARM 1	ARM 2	Overall
**EQ-5D total score (n)**	994	637	1631
Mean (SD)	0.81 (0.17)	0.86 (0.16)	0.83 (0.17)
Median (IQR)	0.84 (0.21)	0.90 (0.19)	0.86 (0.18)
**EQ-5D VAS score (n)**	1001	647	1648
Mean (SD)	71.2 (18.3)	76.6 (16.2)	73.3 (17.7)
Median (IQR)	75 (25)	80 (20)	75 (25.5)
**IBD-DI score (n)**	1000	647	1647
Mean (SD)	30.6 (18.9)	22.3 (16.6)	27.3 (18.5)
**IBD control score, UK only (n)**	29	42	71
Mean (SD)	7.1 (3.7)	7.3 (2.1)	7.2 (2.9)
**WPAI Scores in Total PPIE population**	1001	647	1648
**Work time missed (absenteeism; %)**	517 (52)	324 (50)	841 (51)
Mean (SD)	0.11 (0.25)	0.06 (0.19)	0.09 (0.23)
**Impairment (presenteeism; n)**	484 (48)	315 (49)	799 (48)
Mean (SD)	0.24 (0.21)	0.18 (0.19)	0.22 (0.20)
**Work productivity loss (n)**	484 (48)	315 (49)	799 (48)
Mean (SD)	0.28 (0.24)	0.20 (0.21)	0.25 (0.23)
**Activity Impairment (n)**	602 (60)	368 (57)	970 (59)
Mean (SD)	0.27 (0.24)	0.18 (0.20)	0.24 (0.23)

*EQ-5D* EuroQol-5 dimension, *IBD-DI* inflammatory bowel disease disability index, *IQR* interquartile range, *n* number, *PPIE* patient and public involvement and engagement, *SD* standard deviation, *UK* United Kingdom, *VAS* visual analogue scale, *WPAI* work productivity and activity impairment

**Table 5:** Patient-reported IBD-DI and EQ-5D scores in the PPIE population by country and ARM

	Denmark (n = 31)		France (n = 500)		Germany (n = 335)		Italy (n = 565)		Norway (n = 22)		Poland (n = 214)	
	ARM 1	ARM 2	ARM 1	ARM 2	ARM 1	ARM 2	ARM 1	ARM 2	ARM 1	ARM 2	ARM 1	ARM 2
**EQ-5D total score (n**)	6	11	216	116	124	42	199	116	4	2	85	41
Mean (SD)	0.83 (0.2)	0.95 (0.07)	0.8 (0.2)	0.87 (0.14)	0.83 (0.12)	0.91 (0.1)	0.83 (0.12)	0.91 (0.1)	0.91 (0.04)	0.93 (0.1)	0.84 (0.2)	0.86 (0.15)
Median (IQR)	82.5 (20)	95 (17)	75 (30)	80 (25)	80 (24)	85 (15)	70 (20)	75 (25)	75 (42)	72.5 (15)	80 (35)	75 (15)
**IBD-DI score (n)**	6	11	218	116	124	43	199	119	4	2	87	41
Mean (SD)	18.98 (21.3)	9.58 (10.96)	26.87 (18.41)	17.32 (15.81)	29.73 (18.47)	22.58 (15.48)	35.61 (19.29)	26.92 (18.18)	22.68 (10.87)	18.75 (11.36)	23.64 (20.4)	23.47 (15.52)

	Spain (n = 595)		Turkey (n = 120)		United Kingdom (n = 479)		Romania (n = 118)		Overall (n = 2979)	
	ARM 1	ARM 2	ARM 1	ARM 2	ARM 1	ARM 2	ARM 1	ARM 2	ARM 1	ARM 2
**EQ-5D total score (n)**	224	201	12	45	31	46	93	17	994	637
Mean (SD)	0.8 (0.17)	0.86 (0.16)	0.89 (0.11)	0.92 (0.09)	0.85 (0.13)	0.9 (0.11)	0.84 (0.16)	0.8 (0.15)	0.81 (0.17)	0.86 (0.16)
Median (IQR)	70 (30)	79 (15)	80 (23)	85 (15)	80 (20)	80 (20)	75 (15)	70 (35)	75 (25)	80 (20)
**IBD-DI score (n)**	225	206	12	45	31	4**7**	95	17	1001	647
Mean (SD)	32.93 (18.5)	23.58 (16.79)	30.21 (17.15)	21.51 (13.29)	33.47 (19.42)	16.87 (14.41)	30.54 (16.57)	30.61 (15.71)	30.57 (18.96)	22.3 (16.6)

*EQ-5D* EuroQol-5 dimension, *IBD-DI* inflammatory bowel disease disability index, *IQR* interquartile range, *n* number, *SD* standard deviation, *VAS* visual analogue scale, *WPAI* work productivity and activity impairment

**Table 6:** Patient-reported WPAI scores in the PPIE population by country

	Denmark (n = 31)	France (n = 500)	Germany (n = 335)	Italy (n = 565)	Norway (n = 22)	Poland (n = 214)	Spain (n = 595)	Turkey (n = 120)	United Kingdom (n = 479)	Romania (n = 118)	Overall (n = 2979)
**Work time missed (absenteeism) (n)**	14	157	123	168	3	73	188	17	30	74	847
Mean (SD)	0 (0)	0.08 (0.22)	0.08 (0.21)	0.14 (0.27)	0.04 (0.08)	0.18 (0.31)	0.07 (0.2)	0.03 (0.07)	0.08 (0.13)	0.05 (0.1)	0.09 (0.23)
**Impairment (presenteeism) (n)**	14	150	117	155	3	66	180	17	30	73	805
Mean (SD)	0.06 (0.06)	0.21 (0.21)	0.22 (0.16)	0.26 (0.22)	0.1 (0.17)	0.23 (0.22)	0.2 (0.2)	0.14 (0.14)	0.29 (0.27)	0.18 (0.17)	0.22 (0.2)
**Work productivity loss (n)**	14	150	117	155	3	66	180	17	30	73	805
Mean (SD)	0.06 (0.06)	0.23 (0.23)	0.24 (0.18)	0.29 (0.25)	0.13 (0.23)	0.32 (0.28)	0.22 (0.22)	0.16 (0.17)	0.33 (0.3)	0.21 (0.19)	0.22 (0.2)
**Activity Impairment (n)**	16	198	127	187	4	83	226	29	34	74	978
Mean (SD)	0.06 (0.09)	0.27 (0.25)	0.26 (0.21)	0.28 (0.22)	0.08 (0.15)	0.25 (0.26)	0.21 (0.22)	0.14 (0.19)	0.28 (0.25)	0.17 (0.18)	0.24 (0.23)

*n* number, *SD* standard deviation, *VAS* visual analogue scale, *WPAI* work productivity and activity impairment

## EQ-5D-5L, IBD-DI and WPAI

The EQ5D-5L questionnaire was completed by 994 in ARM 1 and 637 patients in ARM 2. The overall mean total EQ-5D index score was 0.83, with 0.81 and 0.86 in the ARMs 1 and 2, respectively. The EQ-5D scores varied between the countries included in this study. In ARM 1, the highest EQ-5D score was observed in Norway (0.91) and the lowest in France (0.8). In ARM 2, the highest EQ-5D score was observed in Denmark (0.95) and the lowest in Romania (0.8) (Table 5). EQ-5D review of dimensions revealed a greater impact in the pain/discomfort and anxiety depression domains across both arms, whereas it showed a limited impact on mobility, self-care and usual activities.

The IBD-DI questionnaire was completed by 1000 patients in ARM 1 and 647 patients in ARM 2. The overall mean total IBD–DI score was 27.3, with 30.6 and 22.3 in ARMs 1 and 2, respectively (Table 4). The highest IBD–DI scores in ARM 1 and ARM 2 were in Italy (35.6) and Romania (30.6) respectively, whereas the lowest scores were in Denmark (19 and 9.6, respectively; Table 5).

In the PPIE population, 1001 patients ARM 1 and 647 patients in ARM 2, completed the work productivity and activity impairment (WPAI) questionnaire. Work productivity loss was the most affected dimension in both arms, with mean scores of 0.28 and 0.20, respectively (Table 4).

## Discussion

Despite the growing number of cost-effectiveness studies of new pharmacological interventions (biologics), no previous studies have focused on the overall burden of UC, including costs beyond medical management. To our knowledge, this is the first study to adopt a societal perspective at the pan-European level. Compared with indirect costs, the direct medical cost was higher across both arms of the study. Indirect costs, however, were substantial, highlighting the need for a holistic perspective to have a better appreciation on the true cost of care for patients with UC.

Over the years, biologic treatment is the main driver for direct medical costs [15, 17, 18, 21, 22]. It is likely that their proportion in total costs is smaller than what is stated in this study due to the practical difficulties with capturing real transaction prices and also because this study did not capture the proportion of biosimilar use vs cost of original biologic. Accounting for competitive processes such as regional or hospital tenders, non-transparent price discounts etc. poses real practical challenges and whilst desirable, remain unrealistic and must be acknowledged as a possible limitation of our work.

Patients in ARM 1 reported a higher impairment in both HRQoL and work productivity and activity than patients in ARM 2, suggesting a direct relationship between UC activity and morbidity from disease, however a formal statistical comparison was not the objective of this exploratory study. Patients with active UC have been reported to have higher health care related costs from the direct cost of diagnostic tests, medication, hospitalisation and surgery but also from indirect costs such as reduced employment, effect on work-productivity and opportunities for unpaid activities [29–31]. The EQ-5D-5L show a relatively small impact of the disease in patients, with high scores per ARM, which might be explained by low sensitivity of the measuring instrument, the fluctuating nature of the disease (many patients changed to different severity status in the 12 months documentation period, resulting in mixed results) and recall period of 1 day “your health today”. Also, the realistic possibility of corticosteroid therapy in these patients in controlling symptoms but not necessarily achieving meaningful remission may mask true disease activity.

The relatively small differences observed in total costs results in both arms suggest that there are significant unmet needs and associated costs in patients even when they achieve mild and remission status (definition of ARM 2 in this study). Again, the fluctuant nature of disease severity (39% of patients in ARM 1 improving to mild or remission and 23% of those in ARM 2 worsening to moderate or severe) may partly explain these results. The ARM- and country-specific IBD-DI scores imply an impact on Quality of Life, but also suggest that the EQ-5D might not be a sensitive enough tool in a disease with a fluctuating nature such as UC. The mean IBD control score, which was only captured in UK patients, was 7.3 in ARM 2 and 7.1 in ARM 1. Therefore, it is recommended to carry out further detailed analysis of the data, focusing on the relationship between changing severity and clinical and economic outcomes.

The proportion of patients with strictures as a complication of their UC was higher than expected at 12% and 9% for ARM 1 and ARM 2 respectively. Previous studies have reported a prevalence of 1.5%, 3.2% and 6.5% respectively [32–34]. It is possible that this unexpectedly large proportion of UC patients with strictures is indicative of long standing aggressive or sub-optimally controlled disease and is concerning. Alternatively, it is also possible that there has been a degree of misclassification of patients with Crohn’s disease, suggested by the strictures but also by the high prevalence of fistulas in this population (10% and 7% in arms 1 and 2 respectively). Nevertheless, it is important that the study is reflective of everyday practice, where categorisation of patients may not be straightforward.

Also, the proportion of patients experiencing diarrhoea, rectal bleeding, anaemia, crampy abdominal pain and fatigue in ARM 2 was not far behind that in ARM 1, which could explain to some degree the relatively small differences in costs in the two groups. This raises several questions with respect to quality and perception of disease control. It is possible that sub-optimally controlled disease and the naturally fluctuant disease course therein triggered these symptoms and emphasises the need to treat beyond symptoms aiming for endoscopic remission where possible to mitigate gut injury and active symptoms stemming from sub-optimal disease control [35, 36]. The impact of the wider adoption of a treat to target strategy on optimal disease control and its implications to quality of life represent an area of urgent and unmet research need.

There is increasing awareness that ulcerative colitis is a progressive disease and that long standing disease may lead to complications such as stricturing, dysmotility, anorectal dysfunction and altered colonic permeability which may also be a driver of symptoms such as diarrhoea and incontinence [16, 37, 38].

This study had limitations that must be considered before reaching an overall consensus. Some countries (e.g., Spain) have a much larger population sample than others (e.g., Norway), which could increase the uncertainty of the final costs in the latter. EQ-5D results suggest a low sensitivity to changes in disease severity. Additionally, the short recall period "your health today" of this tool, coupled with the fluctuant nature of UC, challenge the interpretation of these data. Despite physicians having no choice in selection of participants, the next ten patients seen in clinical consultation are selected, selection bias may still be present. Mild patients are more likely to be seen in clinical consultation as the disease is often newly diagnosed. Severe patients are also more likely to be seen in clinical consultation as they need the most attention. This can lead to selection bias against remission and/or moderate patients.

The higher costs in the PPIE population compared with those in the CRF population suggests that the responding patients and the main LUCID population should not be compared directly; this difference would partly explain the EQ5D results being higher than expected and suggesting that there might be a difference (bias) between patients who respond versus those who do not.

## Conclusion

The initial results of this study suggest that there is a significant cost associated with UC management in both arms, with a relatively high cost even for patients that reach remission or mild status. Despite the relatively high EQ-5D scores in the UC population, analysis of IBD-DI, work productivity and indirect costs suggest an unmet need in the UC population, especially in ARM 1 of the study. Furthermore, the high level of symptoms, disease complications, comorbidities and EIMs further reinforce this unmet need. This study provided insight into the per-patient costs within 10 participating European countries with markedly different healthcare provisions and settings, thereby aiding stakeholders with the opportunity to understand the economic landscape of the condition better. The outcomes of the LUCID study will help develop relevant public health actions and policies and help improve UC clinical guidelines.

## Supplementary Information

**Additional file 1.** **Supplementary Table 1.** LUCID study cost sources. **Supplementary Table 2.** Number of physicians and patients returned questionnaires.

## Data Availability

The data that support the findings of this study are available from HCD Economics but restrictions apply to the availability of these data, which were used under license for the current study, and so are not publicly available. Data are however available from the authors upon reasonable request and with permission of HCD Economics.

## Abbreviations

UC: Ulcerative colitis
eCRF: Electronic case report forms
HRQoL: Health-related quality of life
PPIE: Public and patient involvement and engagement
IBD: Inflammatory bowel disease
PROMS: Patient reported outcome measures
UoC: University of Chester
GPP: Guidelines for Good Pharmacoepidemiology Practices
ERG: Expert review group
CCUK: Crohns and Colitis UK
WPAI: Work productivity and impairment
EQ-5D: EuroQoL-5D
IBD-DI: Inflammatory bowel disease-disability index
RU: Resource use
SCCAI: Simple clinical colitis activity index
PGA: Physician global assessment
BMI: Body mass index
EIM: Extra Intestinal manifestations
