# Correction to: The LUCID study: living with ulcerative colitis; identifying the socioeconomic burden in Europe

**DOI:** 10.1186/s12876-021-02062-3

**Published:** 2021-12-15

**Authors:** Leonardo Ruiz-Casas, Jonathan Evans, Alison Rose, Gabriel Ghizzi Pedra, Alan Lobo, Alan Finnegan, Bu Hayee, Laurent Peyrin-Biroulet, Andreas Sturm, Johan Burisch, Helen Terry, Luisa Avedano, Seb Tucknott, Gionata Fiorino, Jimmy Limdi

**Affiliations:** 1 HCD Economics, The Innovation Centre, Keckwick Lane, Daresbury, Warrington, England, UK; 2 Sheffield Teaching Hospitals NHS Foundation Trust, Sheffield, UK; 3 The University of Sheffield, Sheffield, UK; 4 University of Chester, Chester, UK; 5 King’s College Hospital NHS Foundation Trust London, London, UK; 6 University Hospital Centre Nancy, Nancy, France; 7 DRK Kliniken Berlin, Berlin, Germany; 8 Hvidovre Hospital, Gastrounit, Hvidovre, Denmark; 9 Crohn’s and Colitis UK, Hatfield, Hertfordshire, UK; 10 European Federation of Crohn’s and Ulcerative Colitis Associations, Brussels, Belgium; 11 IBDrelief, Brighton, UK; 12 Istituto Clinico Humanitas, Milan, Italy; 13 Pennine Acute Hospitals NHS Trust, Manchester, UK

## Correction to: BMC Gastroenterology (2021) 21:456 10.1186/s12876-021-02028-5

After publication of this article [1], the authors reported that the author name “Avedano” was incorrectly written as “Avendano”.

The original article [1] has been updated.
