# Supplementary material for: The LUCID study: living with ulcerative colitis; identifying the socioeconomic burden in Europe
Source: BMC Gastroenterol. 2021 Dec 4;21:456. doi: 10.1186/s12876-021-02028-5 (PMC8645093; doi:10.1186/s12876-021-02028-5)
Supplement: Supplementary file 1 — Additional file 1. Supplementary Table 1. LUCID study cost sources. Supplementary Table 2. Number of physicians and patients returned questionnaires. [file 12876_2021_2028_MOESM1_ESM.docx]

**Supplementary Table 1 LUCID study cost sources**

| Country | Resources |
| --- | --- |
| France | Ameli.fr, sante.gouv.fr, ViDAL.fr |
| Germany | Kbv.de, meinpharmaversand.de, Einheitlicher Bewertungsmaßstab, rote-liste service |
| Italy | trovanorme.salute.gov.it, Ordinary supplement n. 8 to the Official Journal , Tariffa minima degli onorari per le prestazioni medico-chirurgiche, starbene.it drug search |
| Spain | Oblikue e-salud, Agencia española de medicamentos y productos sanitarios, Regional orders by the Ministry of Health |
| UK  US | National Schedule of Reference Costs, the electronic Medicines Compendium, NICE British National Formulary  Medicaid NADAC database (National Average Drug Acquisition Cost), Centre for Medicare and Medicaid service (CMS), Physician fee schedule, American Medical Association |
| All countries | World Health Organisation, OECD statistics ([data.oecd.org](https://data.oecd.org/)), grey literature and reports |

**Supplementary Table 2 Number of physicians and patients returned questionnaires**

| Country | Case Record Forms (CRFs) | Number of physicians (gastroenterologists) | Patient Public Involvement Engagement (PPIEs) returned | Response rate (%) |
| --- | --- | --- | --- | --- |
| Denmark | 31 | 6 | 17 | 55 |
| France | 500 | 63 | 334 | 67 |
| Germany | 335 | 47 | 167 | 50 |
| Italy | 565 | 70 | 318 | 56 |
| Norway | 22 | 3 | 6 | 27 |
| Poland | 214 | 26 | 128 | 60 |
| Spain | 595 | 69 | 431 | 73 |
| Turkey | 120 | 13 | 57 | 48 |
| UK | 479 | 62 | 78 | 16 |
| Romania | 118 | 24 | 112 | 95 |
| All (%) | 2,979 | 379 | 1,648 | 55 |
